# Form of Neuralgia of the Jaw-Bones Hitherto Undescribed

**Published:** 1870-10

**Authors:** S. D. Gross

**Affiliations:** Prof. Surgery, etc.


FORM OF NEURALGIA OF THE JAW-BONES HITHERTO UNDESCRIBED. (*By S. D. Gross, M.D., Prof. Surgery, etc.*) The seat of this affection is in the remnants of the alveolar process of edentulous persons, or in the alveolar structures of the overlying gums, and occurs mainly in elderly persons. It occurs most often in the upper jaw, and usually affects but a small extent of surface. The soft tissues in the immediate vicinity do not seem to suffer as in ordinary neuralgia, save in a slight degree. The morbid action is generally limited to the bone, although the gum may be, in rare instances, involved. The gum is nearly always hard and dense, grating under the knife, and adhering with great firmness to the atrophied alveolar process beneath.

The pain is generally paroxysmal, as in ordinary neuralgia, the slightest causes provoking it, as talking, masticating, the contact of hot or cold fluids, deglutition, or mental excitement. Sometimes it is momentary, occasionally it lasts for hours, and a few cases occur in which it continues with little mitigation for an indefinite period. The pain varies in character, and pressure relieves rather than aggravates it.

The pathology of the affection seems to be compression of the minute nerves distributed through the wasted alveolor process, dependent upon the encroachment of osseous matter upon the walls of the canals in which they are naturally enclosed. The osseous structure is always abnormally hard from the deposit of new substance which imparts to it an almost ivory-like consistence.

The disease usually comes on gradually, growing worse, until the suffering is intolerable. The general health becomes eventually impaired, the appetite deranged, the sleep disturbed, the countenance anxious, digestion imperfect, bowels constipated, and the spirits much depressed. Sometimes the disease would seem to be of malarious origin, the attacks coming on periodically. The professor gives a history of six cases which illustrate this disease, and the treatment he institutes for its relief.

The treatment consists in carrying an incision along the alveolar ridge over the painful structures, dividing and turning aside the soft parts, including the periosteum, and removing, by means of cutting pliers (constructed for the purpose), and the gouge, the affected bone to a level with the palatine process.

He has not found the bone at all diseased, and, from all his operations, recovery has been both prompt and complete.—*Am. Journal of Medical Science.*

